# Safety of spinal anesthesia and analysis of cerebrospinal fluid in SARS-CoV-2 pregnant women undergoing cesarean section: an observational prospective study

**DOI:** 10.1186/s44158-023-00135-1

**Published:** 2023-11-28

**Authors:** Giulia Fierro, Barbara Milan, Silvia Bettinelli, Elisa Bottari, Dario Bugada, Ilaria Roncagliolo, Marco Arosio, Claudio Farina, Ferdinando Luca Lorini

**Affiliations:** 1 Department of Emergency and Intensive Care, ASST Papa Giovanni XXIII, 24127 Bergamo, Italy; 2 Department of Intensive Care Unit, Royal Brompton Hospital, London, UK; 3 Department of Anesthesia and Intensive Care, University of Milan, 20122 Milan, Italy; 4 Microbiology and Virology Laboratory, ASST Papa Giovanni XXIII, Bergamo, Italy; 5 Biobank, ASST Papa Giovanni XXIII, Bergamo, Italy

**Keywords:** Pregnancy, SARS-CoV-2, Spinal anesthesia, Cerebrospinal fluid, Chemical-physical analysis

## Abstract

**Background:**

Systemic infection has always been considered a relative contraindication to neuraxial anesthesia, despite the fact that infectious complications are relatively uncommon. Pregnancy-related physiological changes and coronavirus disease (COVID-19) neurotropic features may facilitate the virus’ entry into the central nervous system. The principal aim of this study was to test the safety of spinal anesthesia in “severe acute respiratory syndrome coronavirus 2” (SARS-CoV-2)-positive pregnant women and to examine cerebrospinal fluid (CSF) characteristics.

**Methods:**

We conducted a prospective observational single-center study in asymptomatic or paucisymptomatic consecutive pregnant SARS-CoV-2 patients who underwent spinal anesthesia for cesarean section. Women with severe infection were excluded because they underwent general anesthesia. At the time of spinal anesthesia, we collected CSF samples, and then we performed a chemical-physical analysis to look for signs of inflammation and for SARS-CoV-2 genome.

**Results:**

We included 26 women. No spinal anesthesia complications were reported in the perioperative period and after 2 months. All CSF samples were crystal clear, and all physical-chemical values were within physiological ranges: the median concentration of CSF/plasma glucose ratio was 0.66, *IQR* 0.5500 (0.6000–0.7100), and the average CSF protein concentration value was 23.2 mg/dl (SD 4.87). In all samples, genomes of SARS-CoV-2 and other neurotropic viruses were not detected.

**Conclusions:**

Spinal anesthesia was safe in SARS-CoV-2 pregnant women with mild disease; no clinical maternal complications were detected, and no CSF changes indicative of inflammatory or infectious diseases that would compromise the safety of the procedure were found.

## Background

Since the beginning of the pandemic, data on the effects of severe acute respiratory syndrome coronavirus 2 (SARS-CoV-2) on pregnant women has been limited [1]. According to the Italian Society of Anesthesia and Intensive Care, there are no contraindications to neuraxial regional anesthesia (NRA) for SARS-CoV-2-positive pregnant women undergoing cesarean section (CS) [2]. NRA is considered the technique of choice to preserve fetal and maternal well-being [3].

To date, no data are available about the safety of performing NRA in pregnant women positive for SARS-CoV-2, regardless of the presence of symptoms. Even though a preexisting infection has always been considered a relative contraindication to NRA, several studies have shown that major problems, like infectious complications, are rare after spinal and epidural anesthesia, even in pregnant women [4–6].

Despite the fact that SARS-CoV-2 patients mostly developed respiratory symptoms, neurotropic features of the virus have been known since the beginning of the pandemic. In fact, SARS-CoV-2 may cause central neurological symptoms and complications by either directly accessing the central nervous system (CNS) or through an inflammatory-cytokine activation (cytokine storm) [7].

Pregnancy leads to several anatomical and physiological changes, and the CNS is also involved [8]. A decrease in cerebrospinal fluid (CSF) volume and an increase in progesterone occur, resulting in a direct effect on membrane excitability, an indirect effect on neurotransmitters, and an increase in neuronal sheath permeability [9]. Modifications also involve the vascular endothelium and blood-brain barrier (BBB) permeability, as well as the physiological mechanism of cerebral flow self-regulation [10].

The CNS physiological changes during pregnancy and the presence of SARS-CoV-2 infection may reduce the safety of NRA.

The principal aim of this study was to test the safety of NRA in SARS-CoV-2 pregnant women undergoing CS and to perform CSF analysis to evaluate the presence of the SARS-CoV-2 genome and chemical-physical signs of inflammation.

## Methods

### Study design and patients

We conducted a prospective observational single-center study in SARS-CoV-2 consecutive pregnant women without severe infection who underwent CS, to test the safety of NRA through the analysis of CSF.

The study is designed and presented according to the STROBE guidelines [11].

Data were collected at the Obstetric Department of ASST Papa Giovanni XXIII (Bergamo, Italy), a tertiary care referral center (Italian Ministerial Decree n. 70/2015) for obstetric care in the North of Italy.

Since the beginning, due to the exceptional pandemic period, the ethical committee approved all the studies on COVID-19, and the release of data was authorized in 2022 (REF: no. 61/2022).

**Fig. 1:**
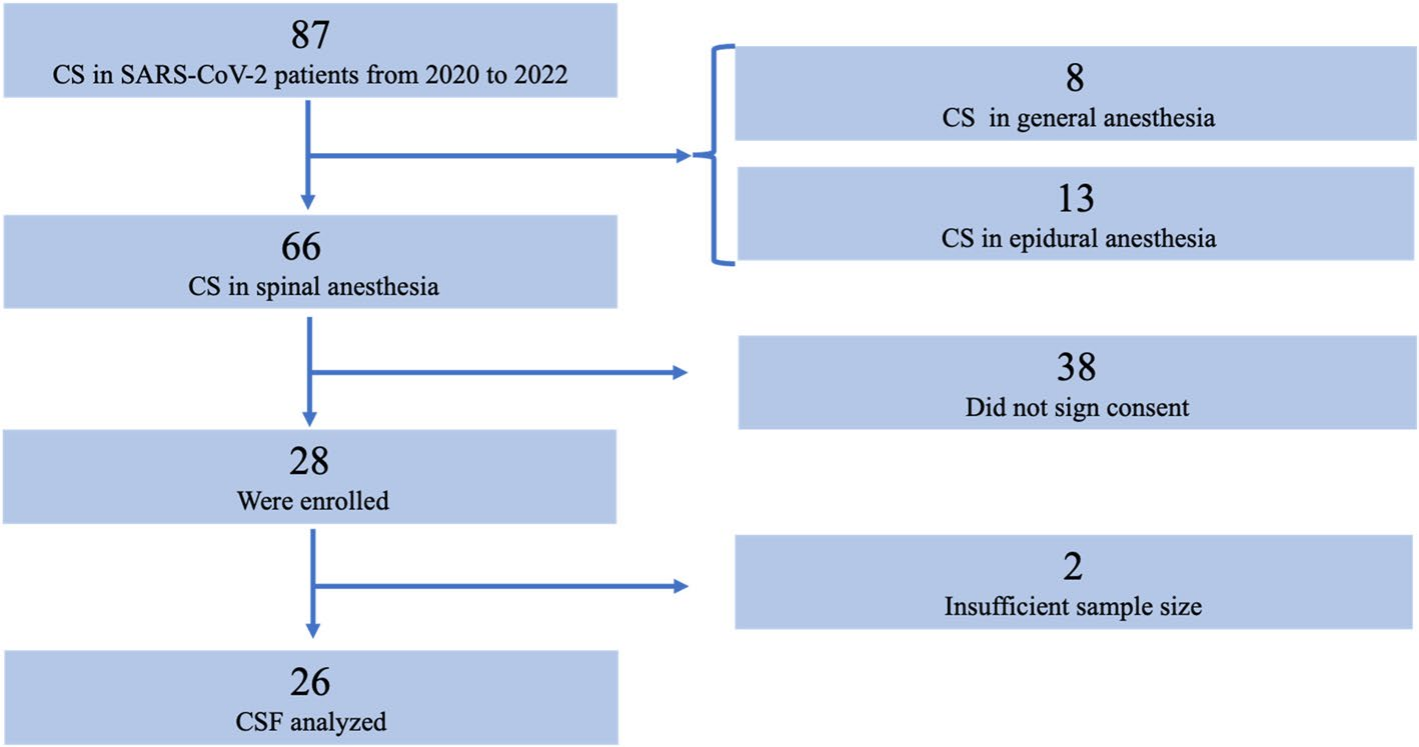
The flow diagram shows the enrolment process. Abbreviations: CS, cesarean section; CSF, cerebrospinal fluid

Patients were recruited between May 2020 and February 2022; the enrollment process is shown in Fig. 1.

Inclusion criteria were asymptomatic or paucisymptomatic SARS-CoV-2-positive pregnant women aged > 18 years who underwent CS under spinal anesthesia and who gave consent for both CSF collection and the subsequent analyses. Exclusion criteria were as follows: age < 18 years, women with severe symptoms (oxygen saturation < 95% with oxygen therapy and/or hemodynamically unstable) because they underwent CS under general anesthesia, women who underwent CS in epidural anesthesia after analgesia in labor, and women who did not give informed consent.

All enrolled patients did not receive antiviral or corticosteroid therapy for the treatment of SARS-CoV-2 infection in the preoperative period since the symptoms were absent or mild SARS-CoV-2 positivity was identified by real-time reverse-transcriptase polymerase-chain reaction (RT-PCR) performed on nasopharyngeal specimens at the time of admission.

For elective CS, admission was scheduled the evening before surgery. Urgent CS was indicated for labor dystocia, and the SARS-CoV-2 test was performed as soon as the mother entered the delivery room.

We recorded demographic and clinical data (age, body mass index, gestational age, parity, elective or urgent CS, vaccination status) and the presence of one of the following symptoms and clinical signs at the moment of NRA: fever, headache, cough, anosmia, dysgeusia, rhinorrhea, diarrhea, and a preoperative chest radiograph (CXR) positive for pneumonia. In addition, a blood test was performed before CS to measure plasma glucose and blood C-reactive protein levels.

A CSF sample was obtained when spinal anesthesia was performed at the time of the CS. In asepsis, a 25-gauge spinal needle was introduced into the lumbar subarachnoid space, and 2.5 ml of CSF was collected prior to intrathecal administration of the anesthetic drugs. Afterwards, each patient received 2.5 ml of levobupivacaine, 0.5% isobaric with sufentanil 2.5 μg in 0.5 ml, and morphine 100 μg in 1 ml for a total volume of 4 ml.

After spinal anesthesia, an intravenous bolus of ephedrine (6 mg) and phenylephrine (50 mcg) was administered to prevent hypotension. This was repeated if necessary.

CSF samples were stored at −80 °C in the hospital biobank [12], and later, chemical-physical analysis and SARS-CoV-2 viral RNA research were performed.

We looked for complications of spinal anesthesia in the perioperative period and, through a phone inquiry, 2 months after CS. We assessed the presence of orthostatic headache, paresthesias, dysesthesias, and myalgias in the lower limbs.

### Chemical-physical analysis

CSF chemical-physical analysis was performed on the Atellica Siemens platform (CH module). The total protein assay was performed with the colorimetric biuret technique (*D* = 600 nm). Glucose levels were determined through the enzymatic hexokinase method. Afterwards, we calculated the CSF/plasma glucose ratio as previously indicated by Bernardi et al. [13]. The C-reactive protein dosage was performed with a high sensitivity turbidimetric method using latex microparticles (hs-PCR). Lastly, CSFs lactate was dosed with a lactate oxidized/peroxidase reaction which produces a violet dye measured by a spectrophotometer. Flow cytometric analysis could not be performed because sample cellularity is affected by the defrosting process of the specimen. All normal ranges were used as a parameter in our analysis referred to the healthy general population values.

### Detection of viruses

All samples were processed for the presence of the SARS-CoV-2 genome (RdRp and ORF8 viral proteins genes). Additionally, each sample was analyzed in order to target specific genomic regions of the following viruses: herpes simplex virus 1, herpes simplex virus 2, Epstein-Barr virus, *Cytomegalovirus*, human herpesvirus 6, varicella-zoster virus, and *Enterovirus*. These tests were performed using the ELITe MGB Real-Time PCR kits in association with the ELITe InGenius platform, according to the manufacturer’s instructions.

### Statistical analysis

Nonnormal distributed data are presented as median and interquartile range (IQR) (25th, 75th percentiles). Normal distribution was assessed using the Shapiro-Wilk test, and qualitative variables are presented as frequencies and percentages. Statistical analysis and plot modeling were performed using SPSS (Version 28).

## Results

Between March 2020 and February 2022, 87 patients scheduled for CS tested positive for SARS-CoV-2. Of these, 66 underwent CS with spinal anesthesia, and 26 were included in the final analysis, according to Fig. 1.

In our population, elective CS was scheduled in 80.8% of the cases, while 19.2% of the women underwent CS in urgent regime. The gestational age at delivery was > 37 weeks in 22 women and between 32 and 37 weeks in 4 patients; 61.5% of women were asymptomatic, and 38.5% were symptomatic.

Detailed demographic and clinical data are summarized in Tables 1 and 2. No spinal anesthesia complications were reported in the perioperative period and after 2 months.

**Table 1:** Demographic date, clinical data, frequency of symptoms, and clinical signs

Demographic data	
Age (*mean*; *SD*)	32.8; 2.99
BMI (*mean*; *SD*)	32.8; 9.18
Parity (*n*; *%*)	Multiparous: 3 (11.5) Nulliparous: 23 (88.5)
Vaccinated (*n*; *%*)	8 (30.7)
**Symptoms at the time of CS**	**Frequency (*n*; %)**
None	16 (61.5)
Fever	7 (26.9)
Headache	1 (3.8)
Anosmia	3 (11.5)
Dysgeusia	3 (11.5)
Rhinorrhea	2 (7.7)
Cough	3 (11.5)
Diarrhea	1 (3.8)
Ground-glass opacity (GGO) at CXR	2 (7.7)

The total percentage is more than 100% as patients could have more than one symptom

*SD* Standard deviation, *BMI* Body mass index, *CS* Cesarean section, *CXR* Chest radiography

**Table 2:** Clinical features COVID-19 related at the time of cesarean section (CS)

Acute clinical features COVID related								
Patient (*n*)	Presence of symptoms							Positive CXR^a^
1-15	None							
16	None							Yes
	Fever	Headache	Anosmia	Dysgeusia	Rhinorrhea	Cough	Diarrhea	
17	Yes		Yes	Yes	Yes			
18			Yes	Yes				
19	Yes		Yes	Yes				
20						Yes	Yes	
21	Yes							Yes
22	Yes							
23	Yes							
24						Yes		
25	Yes							
26	Yes	Yes			Yes	Yes		

^a^CXR Chest radiography

### Chemical-physical analysis

**Fig. 2:**
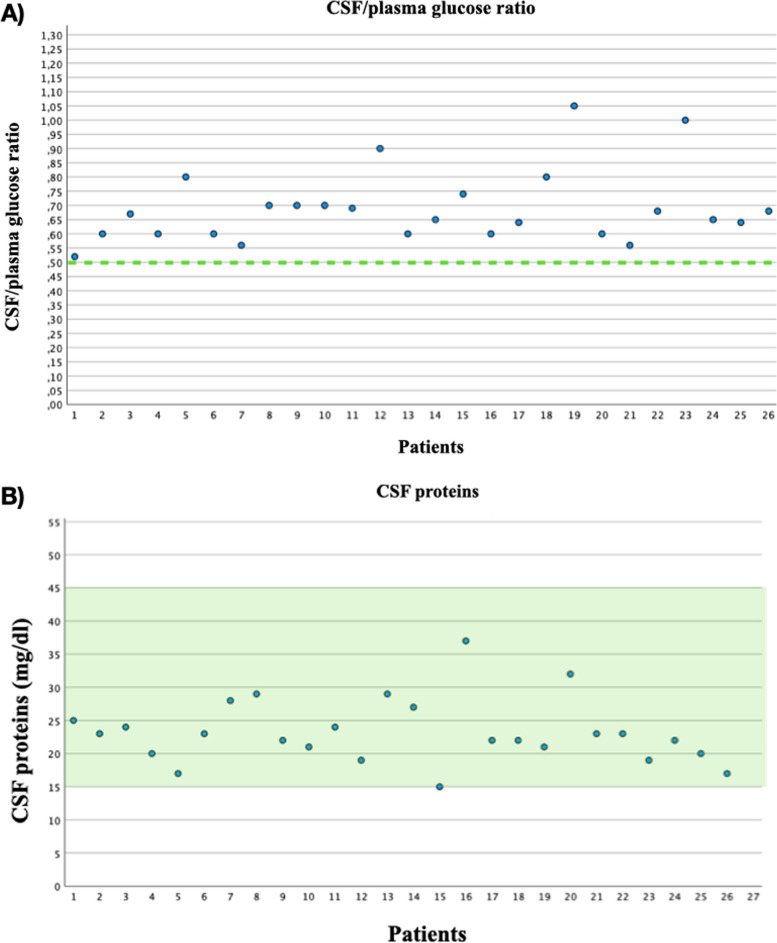
Cerebrospinal fluid (CSF)/plasma glucose ratio and CSF proteins. The dotted line in **A** corresponds to the lower limit of the physiological value (> 0.5). The green area in **B** represents the physiological range (between 15 and 45 mg/dl)

All CSF samples were crystal clear, the median concentration of CSF/plasma glucose ratio was 0.66 with *IQR* 0.5500 (0.6000–0.7100), the average CSF protein concentration value was 23.2 mg/dl, and standard deviation (SD) was 4.87. Data are summarized in Fig. 2.

All CSF C-reactive protein values resulted < 0.05 mg/dl, while the blood C-reactive protein concentration resulted > 1 mg/dl in 20 patients. Only 8 samples were sufficient to perform lactate levels detection; CSF lactate average value was 1.7 mmol/l (*SD* 0.21). All blood and CSF values obtained are summarized in Table 3.

**Table 3:** Blood and CSF (Cerebrospinal Fluid) values

	Blood WBC* 10^**9**^/l	CSF glucose mg/dl	Blood glucose mg/dl	CSF/plasma glucose	CSF protein mg/dl	Blood protein mg/dl	Blood PCR mg/dl	CSF chloride mmol/l
		Physiologic *range 50–80 mg/dl*		Physiologic *value > 0.5*	Physiologic *range 15–45 mg/dl*			Physiologic *range 118–132 mmol/l*
25% percentile	9.430	48.75	72.25	0.6000	20.00	5.100	1.125	119.0
Median	12.38	51.50	77.00	0.6600	22.50	5.550	2.700	120.0
75% percentile	14.97	55.25	81.00	0.7100	25.50	5.825	5.000	121.0
IQR§	13.02	29.00	38.00	0.5500	22.00	2.200	19.60	17.00
Mean	12.33	53.73	77.46	0.6888	23.23	5.504	3.9	119.9
SD^a^	3.736	7.723	8.334	0.1302	4.869	0.5071	4.38	3.166

^a^*SD* Standard deviation, §*IQR* interquartile range

*WBC White Blood Cells

### Detection of viruses

In each CSF sample, the presence of SARS-CoV-2 and other neurotropic viral genomes (herpes simplex virus 1, herpes simplex virus 2, Epstein-Barr virus, *Cytomegalovirus*, human herpesvirus 6, varicella-zoster virus, and *Enterovirus*) were not detected.

## Discussion

Our study showed that NRA is a safe procedure in SARS-CoV-2 pregnant women. We did not find any inflammation signs, and all CSF samples resulted negative for the detection of SARS-CoV-2 RNA.

No previous studies evaluated inflammatory changes in CSF physical-chemical analysis in asymptomatic or paucisymptomatic SARS-CoV-2 pregnant women.

In our analysis, all the CSF samples were crystal clear, and all physical-chemical values were within physiological ranges, even in pauci-symptomatic women. The median concentration of CSF/plasma glucose ratio was 0.66 (physiologic value > 0.5), *IQR* 0.5500 (0.6000–0.7100), and the average CSF protein concentration value was 23.2 mg/dl (physiologic range 15–45 mg/dl) (*SD* 4.87). All physiologic ranges used as a parameter in our study referred to the healthy general population. There is only one study from 1979 that investigated CSF physiologic ranges during physiological pregnancy, but it did not evaluate glucose levels [14].

CSF/plasma glucose ratio and CSF lactate levels are known to be age, but not gender dependent [15]. There are no existing data on their values in pregnancy. Nonetheless, pregnancy could lead to an alteration of these parameters because they are also influenced by gestational age.

CSF lactate concentration does not depend on blood lactate levels; the physiologic range is between 1.2 and 2.1 mmol/l in the adult population [15]. In the 8 analyzed samples, lactate values detected resulted < 2 mmol/l. In the literature, there are no references on CSF lactate values in pregnant women.

The fact that all physical-chemical values were within physiological ranges, despite elevated systemic blood C-reactive protein values, could potentially suggest that the systemic inflammatory process is not predictive of an inflammatory response in CSF. Furthermore, we emphasized that none of the patients received antiviral or anti-inflammatory medication prior to surgery, which may have affected the results of these analyses.

However, it was not possible to assess white blood cells (WBC) count in the CSF, because of cellular lysis due to the sample defrosting procedure.

All CSF samples analyzed were negative for the detection of SARS-CoV-2 RNA. This finding agrees with two previous smaller studies that investigated the presence of viral RNA in the CSF of SARS-CoV-2 pregnant women [16, 17]. In the first one, they enrolled 14 women with mild neurological symptoms, whereas the second is a case report.

Nonetheless, in the literature, the presence of viral RNA in CSF was found only in patients with severe neurological symptoms; in 2021, a review reported an incidence of 6% of SARS-CoV-2 RNA in CSF in patients with severe neurological manifestations [18]. Furthermore, rarely, meningitis in SARS-CoV-2 patients could also be caused by co-infection with other viruses or by autoimmune mechanisms. This is the reason why we also examined the presence of other neurotropic viruses [19]. In our research, all analyzed CSF samples were negative for other viruses.

In our population, none of the patients had intraoperative hypotension, and ephedrine and phenylephrine therapy was effective.

In addition, none had the typical orthostatic CSF headache, despite the collection of CSF, and no complications were reported.

This study has some limitations. Our analyzed cohort was small, and CSF samples were stored; therefore, immediate analysis could not be performed, and cellularity could not be assessed.

Maybe it is too early to demonstrate that NRA is a completely safe procedure in SARS-CoV-2 pauci or asymptomatic pregnant women, although in this study we found that all CSF samples were negative for detection of virus, and also in literature it seems that neurological complications of systemic infections are uncommon.

Currently, CSF analysis appears to be the only clinically acceptable invasive method to evaluate CNS responses to infection. However, the detection rate of SARS-CoV-2 in the CSF is highly dependent on the type of disease and the time of sample collection. The titers of viruses in the CSF may change over the course of a patient’s illness due to possible CSF clearance.

Therefore, CSF testing may fail to give positive results due to delayed sampling. Although some patients showed negative results for SARS-CoV-2 in the CSF, the possibility of CNS infection cannot be completely excluded in these patients, as demonstrated in some autopsy studies. There is a “window of time” for the virus to enter the CNS that needs to be considered [20].

Otherwise, this study contains new ideas that deserve to be explored by other larger sample sizes in multicenter studies and highlights how few references exist for the typical range of CSF values in healthy pregnant patients; as a result, all comparisons were made with reference to the healthy general population.

## Conclusion

Despite the small sample size in our investigation and the limits of the CSF analysis, we discovered no clinical and CSF alterations consistent with inflammatory or infectious conditions that may otherwise compromise the safety of neuraxial procedures, even in SARS-CoV-2 pregnant women.

Certainly, further investigations of CSF features in obstetric patients are necessary. Identification of CSF physiological values in this population is essential for detecting any alterations or biomarkers indicative of obstetrical disorders or systemic pathologies.

## Data Availability

The datasets used and/or analyzed during the current study are available from the corresponding author on reasonable request.

## Abbreviations

BBB: Blood-brain barrier
BMI: Body mass index
CNS: Central nervous system
COVID-19: Coronavirus disease
CS: Cesarean section
CSF: Cerebrospinal fluid
CXR: Chest radiography
GGO: Ground-glass opacity
IQR: Interquartile range
NRA: Neuraxial regional anesthesia
RT-PCR: Real-time reverse-transcriptase polymerase-chain reaction
SARS-CoV-2: Severe acute respiratory syndrome coronavirus 2
SD: Standard deviation
WBC: White blood cells
